# Generation of a homozygous fertilization-defective *gcs1* mutant by heat-inducible removal of a rescue gene

**DOI:** 10.1007/s00497-015-0256-4

**Published:** 2015-02-12

**Authors:** Shiori Nagahara, Hidenori Takeuchi, Tetsuya Higashiyama

**Affiliations:** 1 Division of Biological Science, Graduate School of Science, Nagoya University, Furo-cho, Chikusa-ku, Nagoya, Aichi 464-8602 Japan; 2 JST ERATO Higashiyama Live-Holonics Project, Nagoya University, Furo-cho, Chikusa-ku, Nagoya, Aichi 464-8602 Japan; 3 Institute of Transformative Bio-Molecules (WPI-ITbM), Nagoya University, Furo-cho, Chikusa-ku, Nagoya, Aichi 464-8602 Japan

**Keywords:** Double fertilization, GCS1/HAP2, Heat shock, Cre-*loxP*, Fertilization recovery

## Abstract

**New gametic homozygous mutants.**

In angiosperms, a haploid male gamete (sperm cell) fuses with a haploid female gamete (egg cell) during fertilization to form a zygote carrying paternally and maternally derived chromosomes. Several fertilization-defective mutants in *Arabidopsis thaliana*, including a *generative cell-specific 1* (*gcs1*)/*hapless 2* mutant, the sperm cells of which are unable to fuse with female gametes, can only be maintained as heterozygous lines due to the infertile male or female gametes. Here, we report successful generation of a *gcs1* homozygous mutant by heat-inducible removal of the *GCS1* transgene. Using the *gcs1* homozygous mutant as male, the defect in gamete fusion was observed with great frequency; in our direct observation by semi-in vivo fertilization assay using ovules, 100 % of discharged sperm cells in culture failed to show gamete fusion. More than 70 % of ovules in the pistil received a second pollen tube as attempted fertilization recovery. Moreover, *gcs1* mutant sperm cells could fertilize female gametes at a low frequency in the pistil. This strategy to generate homozygous fertilization-defective mutants will facilitate novel approaches in plant reproduction research.

**Electronic supplementary material:**

The online version of this article (doi:10.1007/s00497-015-0256-4) contains supplementary material, which is available to authorized users.

## Introduction

In angiosperms, male and female gametes are generated by meiosis from pollen and embryo sac mother cells, respectively. The number of sets of chromosomes is reduced to half the original number during meiotic division, resulting in haploid reproductive cells. The male haploid cells, the microspores, divide asymmetrically to form vegetative cells containing the generative cell and develop into male gametophytes, pollen. In angiosperms with tricellular pollen, including *Arabidopsis thaliana*, the generative cell undergoes mitosis to generate two male gametes, i.e., sperm cells, within the pollen. On the other hand, the female haploid cell, the megaspore, undergoes three rounds of mitosis and cellularization to form the embryo sac containing two female gametes, i.e., an egg cell and a central cell. The two sperm cells are delivered by a pollen tube elongated from the pollen by tip growth and released into a female gametophyte. One sperm cell fertilizes the egg cell and the other fertilizes the central cell to form a zygote and a nutritious endosperm, respectively (for a review, see Hamamura et al. 2012). The number of sets of chromosomes is doubled in the zygote by fusion of the paternal and maternal chromosomes of the sperm and egg cells.

Successful double fertilization requires various male–female interactions. Precise pollen tube guidance and reception are critical for sperm cell delivery. These steps are controlled by accessory cells of the female gametes, i.e., two synergid cells, which attract the pollen tube to the embryo sac by diffusible attractant peptides (for a review, see Takeuchi and Higashiyama 2011), recognize pollen tube arrival, and induce pollen tube rupture (for a review, see Kessler and Grossniklaus 2011). After pollen tube discharge, two sperm cells remain in place between the egg and central cells, and these are activated in response to egg-secreted EC1 peptides (Sprunck et al. 2012). Sperm cells possess the plasma membrane proteins GAMETE EXPRESSED 2 (GEX2) for gamete adhesion (Mori et al. 2014) and GENERATIVE CELL-SPECIFIC 1 (GCS1)/HAPLESS 2 (HAP2) (Mori et al. 2006; von Besser et al. 2006) for gamete fusion. Successful molecular recognition between male and female gametes results in double fertilization, and two male nuclei begin to move precisely toward the nuclei of their respective female target cells (Hamamura et al. 2011). Calcium spikes (Hamamura et al. 2014; Denninger et al. 2014) and actin-based male nuclear migration (Ohnishi et al. 2014; Kawashima et al. 2014) in the female gametes are also involved in double fertilization. Once double fertilization is completed in both female gametes of an ovule, pollen tube guidance to the ovule stops in order to block polytubey (Kasahara et al. 2012; Beale et al. 2012; Maruyama et al. 2013; Völz et al. 2013). When *gcs1/hap2* sperm cells are delivered but fail in gamete fusion, a second pollen tube is additionally attracted (Kasahara et al. 2012; Beale et al. 2012). Fertilization recovery by the second pollen tube was shown to increase the fertilization success rate (Kasahara et al. 2012).

To determine the mechanisms of plant fertilization, including the fertilization recovery system, physiological analyses using fertilization-defective mutants have been performed. However, several gametophytic mutants never generate homozygous offspring because half of the haploid male or female gametophytes carrying a mutant allele are unable to complete the reproductive process. These mutants must therefore be maintained as heterozygous lines. For example, no homozygous mutants of *gcs1/hap2* have been reported due to the lack of paternal transmission (Mori et al. 2006; von Besser et al. 2006). The male germ cell-specific transcription factor DUO POLLEN 1 (DUO1) is crucial for regulating sperm cell differentiation and function (Durbarry et al. 2005; Rotman et al. 2005) by activating the expression of many sperm-expressed genes, including *GCS1* (Brownfield et al. 2009; Borg et al. 2011). Therefore, homozygous *duo1* mutants have not been obtained by self-crossing. The lack of the availability of homozygous mutants for essential genes in fertilization interferes with analyses of double-fertilization mechanisms and the fertilization recovery process, as the mutant phenotype in heterozygous mutants is always mixed with the wild-type phenotype. Moreover, large-scale gene expression analyses, such as transcriptomics or proteomics, which are powerful means of identifying novel fertilization-related factors (Qin et al. 2009; Grobei et al. 2009; Okuda et al. 2013), are hampered due to the presence of the wild-type gametophytes. Generation of homozygous mutants would significantly contribute to plant reproduction research.

In mammals, conditional knockout, such as Cre-*loxP*-mediated site-specific DNA recombination, which has also been shown to function in *Arabidopsis* (Russell et al. 1992), is applicable to bypass embryonic lethality by removable gene rescue (for a review, see Kos 2004). Therefore, we examined the application of this system to generate homozygous mutants with defects in fertilization by bypassing the process of fertilization with the expression of a heat-induced removable transgene. Here, we report the generation of *gcs1* homozygous (*gcs1/*−) plants by the heat-inducible Cre-*loxP* recombination system in *A. thaliana*. The phenotypes of *gcs1* homozygous plants were examined to determine the potential utility of this mutant and the associated technology for plant reproduction research.

## Materials and methods

### Plant materials and growth conditions

The *A. thaliana* Columbia (Col-0) accession was used as a wild-type control. Transgenic plants possessing *HTR10p::HTR10:mRFP* (Ingouff et al. 2007) and *RPS5Ap::H2B:GFP* gene, which express histone H2B (At1g07790) fused with GFP by *RIBOSOMAL PROTEIN SUBUNIT 5A* (*RPS5A*; *At3g11940*; Adachi et al. 2011) promoter, were used to visualize sperm cell nuclei and female gametophytic cell nuclei, respectively. Sperm cell nuclei of *gcs1* heterozygous mutants (*gcs1/*+; SALK_135496; Mori et al. 2006) were labeled with the *HTR10p::HTR10:mRFP* transgene.

*A. thaliana* seeds were sterilized with a solution containing 2 % Plant Preservative Mixture™ (Cosmo Bio, Japan), 50 µg/mL magnesium sulfide, and 0.1 % Tween overnight. The seeds were sown on MS medium consisting of 1 × Murashige and Skoog salt (Wako, Osaka, Japan), 2 % sucrose, 1 × Gamborg’s vitamin solution (Sigma, St. Louis, MO), and 0.3 % Gelrite (Wako) and adjusted to pH 5.7 with KOH. For the selection of *gcs1/*+ seedlings, MS medium containing 50 mg/L kanamycin sulfate and 0.8 % Bacto™ Agar (Becton, Dickinson and Company, Le Pont de Claix Cedex, France) instead of Gelrite was used. Plants were germinated and grown in a growth chamber at 22 °C with continuous lighting after cold treatment at 4 °C for 2–3 days. Two-week-old seedlings were transferred to soil and grown at 22 °C with continuous lighting. Hyponex 6-10-5 (Hyponex Japan, Osaka, Japan) was used as fertilizer (1:1,000 dilution).

### Generation of constructs and plant transformation

**Fig. 1:**
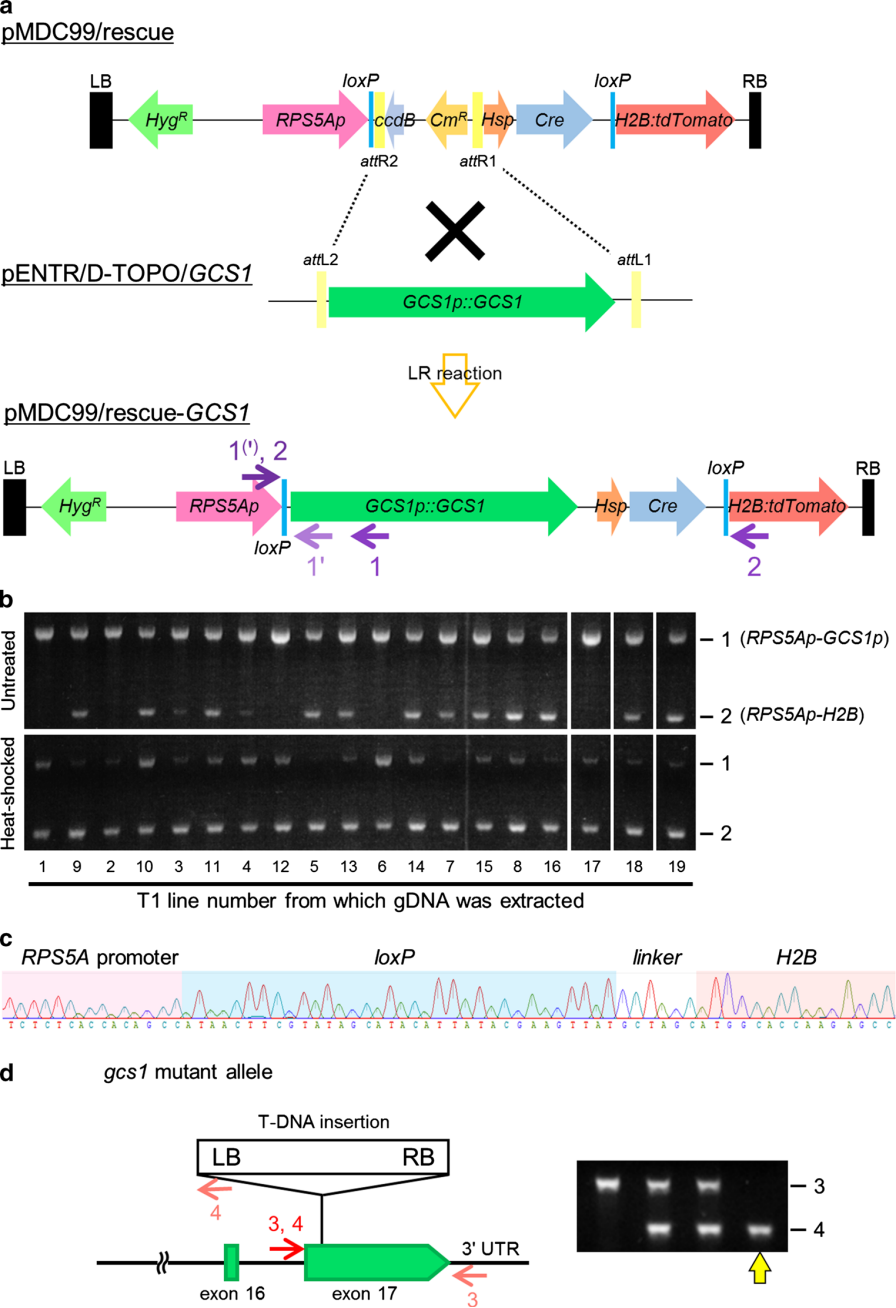
Generation of *gcs1* homozygous mutant rescued by heat-inducible removal of the *GCS1* transgene. **a** Flow chart of pMDC99/rescue-*GCS1* vector construction. The *GCS1p::GCS1* genomic fragment was introduced into the pMDC99/rescue vector by LR reaction. LB, left border region of T-DNA; RB, right border region of T-DNA; *Hyg* ^*R*^, hygromycin resistance gene; *ccdB*, *ccd*B resistance gene; *Cm* ^*R*^, chloramphenicol resistance gene. *Purple arrows* with *numbers* indicate different primer sets used for genomic PCR of the transgenic plants. **b** Genomic PCR of T1 plants. The *upper and lower images* show the gel images of the PCR analysis with non-heat-treated and heat-shocked leaves, respectively. The *numbers* under *each lane* indicate each T1 line number from which genomic DNA was extracted. The *numbers* 1 and 2 on the right side of the gel images indicate the fragments *RPS5Ap*-*GCS1p* and *RPS5Ap*-*H2B*, respectively, amplified with primer sets 1 and 2, described in Fig. 1a. **c** Sequence analysis of the recombined *RPS5Ap*-*H2B* gene. The fragment amplified with primer set 2 in Fig. 1b was confirmed to have the expected *RPS5Ap*-*H2B* sequence with one *loxP* sequence remaining. A part of the analyzed sequence is shown. The *letters* under *each wave* indicate DNA base sequences. **d** Genomic PCR of T2 plants to check whether *gcs1* homozygous plants are produced by the introduced pMDC99/rescue-*GCS1* vector. In the *left image*, the sites of primers are shown by *red arrows* with *numbers*. Primer set 3 amplifying the wild-type *GCS1* gene was designed in intron 16 and the 3′ UTR region to amplify only the endogenous *GCS1* gene. Primer set 4 was designed in the LB (left border) region of T-DNA to detect T-DNA insertion disrupting the *GCS1* gene (*gcs1* allele). The *right image* shows a part of the gel image of genomic PCR of the T2 #19 siblings. The *yellow arrow* indicates the band pattern of the *gcs1/*− plant

To rescue the infertility of sperm cells in *gcs1/*+ mutants, a construct was designed as shown in Fig. 1 (see Table S1 for primers used for construction). Three fragments for *Hsp::Cre*, *loxP*-*H2B:tdTomato*, and *RPS5Ap::loxP* were amplified from a vector (DKv137; pMDC100/*Hsp::Cre*-*RPS5Ap::loxP*-*GUS*-*loxP*-*H2B*-*tdTomato*-*NosT*), which was a kind gift from Dr. Daisuke Kurihara (Nagoya University), and subcloned into pT7Blue vector (Novagen, Madison, WI) or pCR-Blunt II-TOPO vector (Invitrogen, Carlsbad, CA). The *Hsp::Cre* fragment consisted of the promoter region of the gene encoding soybean heat-shock protein [*Gmhsp 17.3*-*B* (Schöffl et al. 1984; Kurup et al. 2005); hereinafter called *Hsp*], an intron-containing sequence of the *Cre*-*int* gene (Zuo et al. 2001), and a *Nos*-terminator sequence. The *loxP*-*H2B:tdTomato* fragment consisted of a *loxP* sequence followed by the histone *H2B* coding sequence of *A. thaliana*, the *tandem dimeric Tomato* (*tdTomato*) gene sequence, and the *Nos*-terminator sequence. The *RPS5Ap::loxP* fragment consisted of the *RPS5A* promoter and a *loxP* sequence. The fragments *RPS5Ap::loxP*, *Hsp::Cre*, and *loxP*-*H2B:tdTomato* were cloned into the pMDC99 cloning vector (Curtis and Grossniklaus 2003) in this order using *Spe*I/*Pac*I sites, *Asc*I/*Kpn*I sites, and *Sbf*I/*Pme*I sites, respectively, resulting in pMDC99/rescue vector. A genomic sequence containing 1,002 bp of upstream and coding regions of *GCS1* gene (*At4g11720*) was amplified using wild-type genomic DNA as a template. The sequence was cloned into the pENTR™/D-TOPO vector (Invitrogen), and the *Nos*-terminator sequence was added downstream of the *GCS1* gene using *Not*I and *Nco*I sites. The *GCS1* fragment was introduced between *RPS5Ap::loxP* and *Hsp::Cre* sequences in the pMDC99/rescue vector by LR reaction using Gateway LR Clonase II Enzyme mix (Invitrogen), resulting in pMDC99/rescue-*GCS1* vector.

The construct was introduced into *Agrobacterium tumefaciens* stain GV3101 (pMP90) by electroporation. The T-DNA construct was transformed into *gcs1/*+ plants by the floral dip method, and transformed seeds were selected on MS medium containing 25 mg/L hygromycin B and 50 mg/L kanamycin sulfate. To confirm the introduction of the T-DNA into the resistant T1 plants, genomic PCR was performed to amplify part of the *Cre* sequence using genomic DNA of each T1 plant as the template. T2 seeds were selected on MS medium containing 25 mg/L hygromycin B or 50 mg/L kanamycin sulfate. The ratio of kanamycin-resistant and kanamycin-sensitive seedlings was counted and subjected to Chi-squared analysis based on an expectation of 50 %. The genomic DNA from hygromycin-resistant T2 plants was extracted and checked by genomic PCR using the primer sets listed in Table S1 to determine whether *gcs1* homozygous mutant plants were produced by the introduced T-DNA. The primers were designed to target the endogenous *gcs1* mutant allele and wild-type *GCS1* allele without amplifying the *GCS1* gene on the introduced T-DNA (Fig. 1d).

### Heat-shock treatment for induction of the Cre-*loxP* system

For T1 plants, heat treatment was performed by exposing leaves collected in 1 mL of sterilized water of a 48-well plate to a temperature of 37 °C for 3 h. The heat-shocked leaves were then incubated for 24 h in a growth chamber at 22 °C, and genomic DNA was extracted from the leaves. To determine whether the Cre-*loxP* system was induced by heat-shock treatment, DNA sequences specific to non-excision (*RPS5Ap*-*GCS1p*) or excision (*RPS5Ap*-*H2B*) of a region between two *loxP* sites were amplified from genomic DNA with the primer sets listed in Table S1.

For heat-shock treatment of T3 plants, which were confirmed to be homozygous for the *gcs1* mutant allele, seeds in 1.5-mL tubes containing sterilization solution or 2- or 7-day-old seedlings sown on MS medium were placed in an incubator at 37 °C for 6 h at each stage. The plants were then returned to the growth chamber at 22 °C and transferred to soil 2 weeks after sowing.

### Expression analysis of *GCS1* by RT-PCR

Total RNA was isolated from the anthers of 30 flowers for both wild-type and T4 *gcs1/*− plants as described below (Fig. 4) using an RNAqueous-Micro Kit (Life Technologies, Gaithersburg, MD) according to the manufacturer’s protocol. Reverse transcription reaction was conducted with a High Capacity RNA-to-cDNA Kit (Life Technologies) according to the manufacturer’s protocol. The amount of cDNA was approximately equalized between the cDNA from wild-type and *gcs1/*− based on the expression level of *TUBULIN BETA CHAIN 4* (*TUB4*; *At5g44340*). The primers were designed to amplify the region upstream of the T-DNA insert and the region sandwiching the T-DNA insert. The primers used are listed in Table S1.

### Semi-in vivo fertilization assay

Semi-in vivo fertilization assay was performed as described previously (Palanivelu and Preuss 2006; Hamamura et al. 2014). Briefly, 150 µL of pollen growth medium was poured into a silicon rubber well on a coverslip. Flowers were emasculated 12–20 h before hand pollination. The pistils of the *RPS5Ap::H2B:GFP* transgenic plants were pollinated by transgenic plants with *HTR10p::HTR10:mRFP*. The hand-pollinated pistils were cut with a 27-gauge needle at the junction between the style and ovary, and the stigmas were then placed horizontally on the pollen growth medium under a stereomicroscope. Ovules from the remaining ovaries were picked out and placed on the medium. Five to six ovules were arranged around the cut end of the stigmas with an insect pin 0.5 mm in diameter. The growth medium in the well was covered with another coverslip and incubated at 22 °C in the dark for about 6 h.

Microscopy settings and image processing for live-cell imaging of semi-in vivo fertilization were performed as described previously (Maruyama et al. 2013). Briefly, about 6 h after pollination (HAP), time-lapse and *z*-plane images were acquired every 5 min and seven planes (3-μm intervals) using a confocal microscope system. Sequential images were acquired after every 100 ms of exposure time. Images were processed with Metamorph version 7.7.7.0 (Universal Imaging Corp., Downingtown, PA) to create maximum-intensity projection images and to add color. Adobe Photoshop CS6 (Adobe Systems, Inc., San Jose, CA) was used to adjust the images. The images and movies were edited using MacBiophotonics ImageJ software (http://www.macbiophotonics.ca/).

### Physiological analyses of ovules and pollen tubes

To examine seed development in pistils pollinated with wild-type or *gcs1/*− pollen, the pistils were cleared after the removal of the ovary wall. The pistils with ovules were dipped into a drop of clearing solution (8:1:3 w/v mixture of chloral hydrate, glycerol, and water) on glass slides and covered with a coverslip. The glass slides with samples were placed into a dark chamber at 4 °C for 3 days and observed using an upright microscope (Axio Imager. A2; Zeiss, Oberkochen, Germany).

To visualize pollen tubes entering the micropyle of ovules in the pistil, aniline blue staining was performed 24 HAP by the method described previously (Hülskamp et al. 1995). Briefly, one side of the ovary wall was removed, and the pistil was fixed on the glass slide with grease. The bare ovules were opened and fixed to each side with grease. The growing pollen tubes on the septum surface and ovules were stained by drops of aniline blue solution (5:8:7 v/v mixture of 2 % aniline blue, 1 M glycerol, pH 9.5, and water). Then, the glass slide was covered with a coverslip, and the sample was immediately observed using a confocal microscope (LSM780-DUO-NLO; Zeiss) or an upright microscope (DP71; Olympus).

## Results

### Generation of *gcs1* homozygous mutant rescued by heat-induced removable *GCS1* transgene

To obtain homozygous loss-of-function mutants for genes essential for gametophytic functions, we generated a rescue construct, pMDC99/rescue, in which introduced sequences of interest can be removed by the heat-induced Cre-*loxP* recombination system (Fig. 1a). This construct was designed to be applicable for the introduction of variable sequences using different entry vectors and site-specific recombination using GATEWAY technology. The *GCS1p::GCS1* genomic fragment was integrated into the pMDC99/rescue vector to complement the *gcs1* defect, resulting in pMDC99/rescue-*GCS1* vector containing *GCS1p::GCS1* and *Cre* recombinase genes between the *loxP* sites (Fig. 1a). We introduced the T-DNA region of the pMDC99/rescue-*GCS1* vector into *gcs1/*+; *HTR10p::HTR10:mRFP* plants and obtained 19 T1 plants. Next, genomic PCR for heat-treated leaves from the T1 plants was performed to investigate whether the Cre-*loxP* recombination system in this construct could work in a heat-inducible manner (Fig. 1b). PCR using primer pairs specific to non-excision (*RPS5Ap*-*GCS1p*; primer set 1 in Fig. 1a) or excision (*RPS5Ap*-*H2B*; primer set 2 in Fig. 1a) of a region between two *loxP* sites showed that all of the heat-shocked T1 leaves had the *RPS5Ap*-*H2B* sequence produced after *loxP* recombination (Fig. 1b). We also confirmed that the fragment amplified with primer set 2 corresponded to the *RPS5Ap*-*H2B* sequence with the *loxP* sequence between the *RPS5Ap* and *H2B* sequences as expected (Fig. 1c). In 14 of 19 T1 lines, amplification of the recombinant *RPS5Ap*-*H2B* sequence was detected in genomic DNA from non-heat-treated leaves, indicating that leaky expression of Cre recombinase by *Hsp* promoter led to spontaneous recombination. Amplification of the *RPS5Ap*-*GCS1p* sequence was decreased by heat treatment in almost all T1 plants, indicating that the Cre-*loxP* recombination system in the construct functioned properly under the control of the *Hsp* promoter in leaves.

To confirm whether the introduced construct rescued the infertile phenotype of *gcs1* sperm cells, we performed segregation analysis of T2 seeds obtained from 12 T1 lines using the kanamycin resistance cassette linked to the *gcs1* mutant allele (Mori et al. 2006). We found a significant increase in kanamycin-resistant plants compared to the expected value (50 %) from self-pollinated heterozygous *gcs1/*+ (Table 1), indicating that the *gcs1* allele was paternally transmitted by the rescue of the infertile phenotype in T1 plants. T2 lines with single insertion of the introduced T-DNA were also chosen by segregation analysis of hygromycin resistance (Table 1). Finally, we selected plants homozygous for the *gcs1* allele by genomic PCR in T2 hygromycin-resistant plants from T1 lines #3, 6, 12, 16, and 19 (Fig. 1d).

**Table 1:** Segregation analysis of T2 plants and heat-shock treatment in T3 generation

T1 line number	Kan^R^ (%)	*n*	Hyg^R^ (%)	*n*	T2 line number	Appearance ratio of infertile plants^a^		
						Condition of heat treatment		
						37 °C; 2 and 7d	37 °C; 0, 2 & 7d	42 °C; 0, 2 & 7d
#1	41.7	60	50.9	55	ND^b^			
#2	57.6	165	69.6	184				
#3	66.3**	163	76.6	171	#3-1	1/4 (0/1)	2/3 (1/2)	ND
					#3-10	0	ND	ND
					#3-14	1/1 (1/1)	5/6 (5/5)	4/4 (3/4)
					#3-20	1/5 (0/1)	2/2 (1/2)	1/2 (0/1)
#4	56.8	37	89.3	56	ND			
#5	57.9	38	100	45				
#6	67.3**	165	77.1	170	#6-5	0/5	ND	ND
					#6-8	0/6		0/3
					#6-9	0/4		ND
					#6-10	0/6		0/2
					#6-14	0/6		0/4
					#6-20	0/4		ND
					#6-23	0/6		0/1
#7	46.4	56	77.8	54	ND			
#10	47.4	57	71.2	52				
#12	74.4**	180	84.39	173	#12-3	0/5	ND	0/4
#13	56.9	58	83.3	60	ND			
#16	70.1**	174	79.2	173	#16-5	0/6	ND	0/4
					#16-10	0/6	1/2 (1/1)	3/4 (2/3)
					#16-19	2/6 (0/2)	5/5 (5/5)	ND
					#16-23	1/6 (0/1)	6/6 (5/6)	1/2 (0/1)
#19	64.6*	164	83.1	172	#19-3	0/6	ND	0/2
					#19-12	0/6	6/6 (5/6)	1/1 (1/1)
					#19-16	0/5	5/5 (5/5)	1/1 (0/1)
					#19-19	1/5 (0/1)	1/3 (1/1)	ND
WT	ND	ND	ND	ND	WT	0/6	0/4	ND
*gcs1/*+	52.4	212	ND	ND	*gcs1/*+	0/2	0/9	0/1

An asterisk (*) and double asterisks (**) indicate significant differences by Chi-squared analysis based on an expectation of 50 % of kanamycin-resistant (Kan^R^) seedlings, which is expected from self-pollinated heterozygous *gcs1/*+

* *p* < 0.05; ** *p* < 0.01. Single insertion of the introduced T-DNA was also predicted by segregation analysis of hygromycin resistance (Hyg^R^) based on an expectation of 83 %

^a^The appearance ratio of infertile plants in the heat-shocked T3 generation is shown. Both fully and partially infertile plants were included. The numbers in parentheses indicate the ratio of the fully infertile plants in the total infertile plants

^b^ *ND* not determined

### Infertile transgenic *gcs1* mutant plants can be induced by heat-shock treatment

**Fig. 2:**
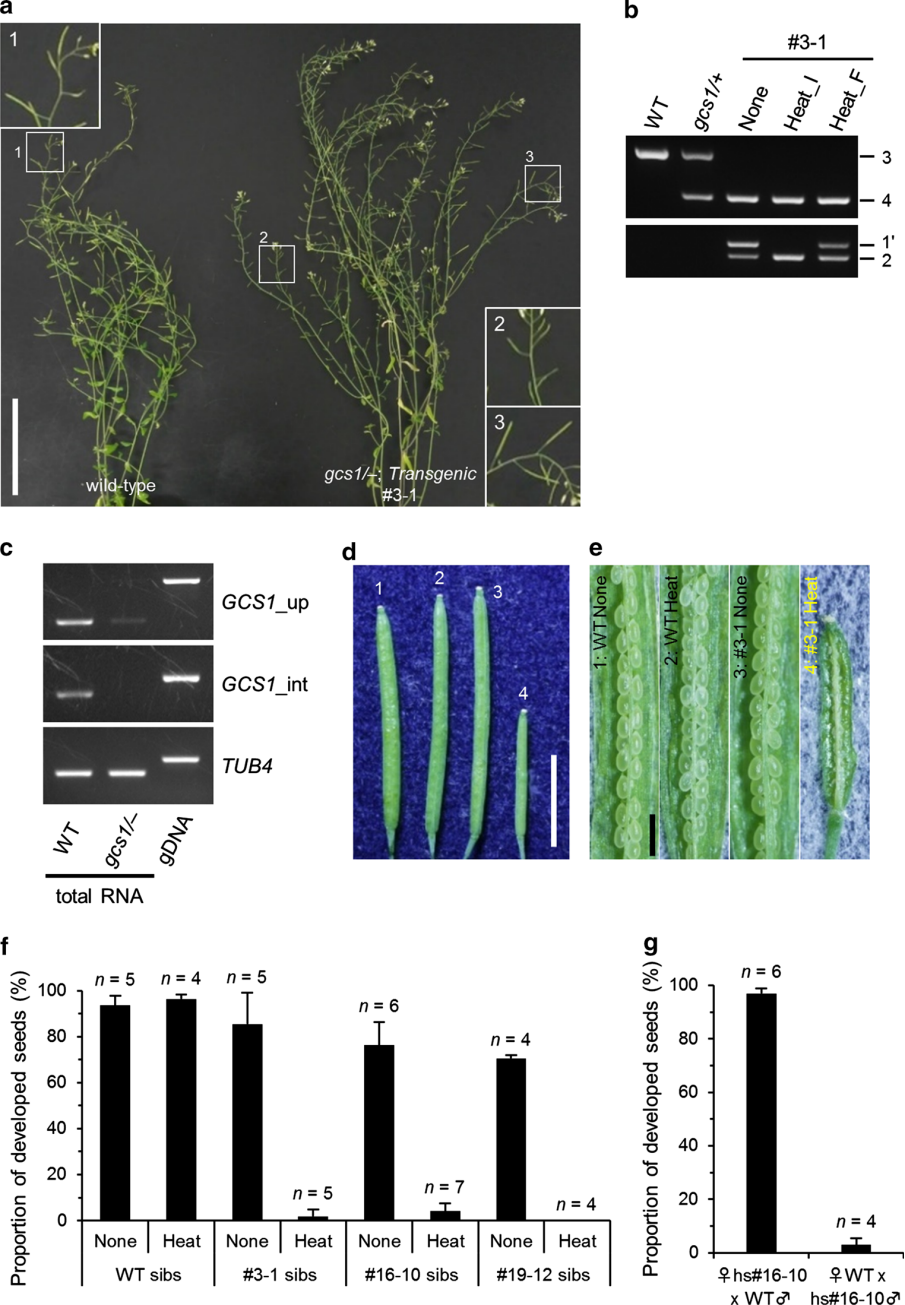
Genotype and phenotype analyses of heat-shocked infertile plants. **a** Comparison of the heat-shocked wild-type plant (*left*) with the heat-shocked T3 #3-1 plant (*right*). *Insets* show magnifications of the fertile (1, 3) and infertile (2) siliques in areas marked by *squares *with *numbers*. *Scale bar*, 10 cm. **b** Genomic PCR of T3 plants to confirm complete removal of *GCS1*. The endogenous *GCS1* (*upper*) and exogenous *GCS1* on introduced T-DNA (*lower*) were amplified with primer sets 3 and 4 shown in Fig. 1d and primer sets 1′ and 2 shown in Fig. 1a, respectively. Genomic DNA was extracted from wild-type (WT), *gcs1/*+, and #3-1 siblings. Genomic DNA of #3-1 siblings was extracted from leaves of the non-heat-treated plant (None), infertile (Heat_I), and fertile (Heat_F) branches of the heat-shocked plant. **c** RT-PCR analysis of the *gcs1/*− plants. Total RNA isolated from wild-type plant or T4 *gcs1/*− plant (Fig. 4) and genomic DNA were used as PCR templates. *Target genes* are indicated on the right side of each gel image. *GCS1*_up and *GCS1*_int indicate the region upstream of the T-DNA insert and the region interrupted by the T-DNA insert in the *gcs1* allele, respectively. **d** Seven days after pollination (DAP) wild-type siliques crossed with untreated (1) or heat-shocked (2) wild-type plants and untreated (3) or heat-shocked (4) T3 #3-1 plants. *Scale bar*, 5 mm. **e** Seed development in each silique shown in Fig. 2d. *Scale bar*, 1 mm. **f** Quantitative analysis of the proportion of developed seeds in the seven DAP siliques pollinated by untreated and heat-shocked wild-type and independent T3 lines (#3-1, #16-10, #19-12). *Error bars* indicate standard deviation. *n*, Number of siliques examined. **g** Reciprocal crossing of heat-shocked #16-10 plant with wild-type plant. The proportion of developed seeds in the seven DAP siliques was counted. *Error bars* indicate standard deviation. *n*, Number of siliques examined

To generate plants lacking both endogenous and transferred *GCS1* by the heat-inducible Cre-*loxP* recombination system, we attempted to define the optimal heat treatment conditions using T3 seeds that were confirmed homozygous for *gcs1* in the T2 generation (Table 1). As the heat-inducible recombination system was not expected to function effectively within the shoot apical meristem forming the aerial part of the plant body, seeds and/or seedlings were heat-treated several times (see Methods). When T3 seedlings were treated at 0, 2, and 7 days old for 6 h at 42 °C in accordance with the method reported previously (Kurup et al. 2005), most treated seedlings whitened and no longer grew, although some of the heat-shocked seedlings survived and showed infertility (Table 1). Therefore, we modified the heat treatment conditions to 37 °C twice at 2 and 7 days old to reduce heat damage. Some plants from lines #3, 16, and 19 had markedly short siliques with no or few seeds (Fig. 2a; Table 1). However, most showed partial infertility with long fertile siliques in some branches. When exposed to 37 °C treatment at 0 days old (i.e., seed stage) in addition to 2 and 7 days old, the majority of the heat-shocked plants exhibited the fully infertile phenotype, in which all branches had sterile siliques, whereas wild-type plants did not show this infertile phenotype (Table 1). Based on these results, we concluded that three rounds of 37 °C heat treatment of transgenic plant seedlings at 0, 2, and 7 days old efficiently generated heat-shock-dependent fully infertile plants.

To confirm whether the infertility resulted from complete removal of the *GCS1* transgene, we performed genomic PCR using cauline leaves from non-heat-shocked fertile and heat-shocked infertile #3-1 sibling plants (Fig. 2a, b). As we found a partially fertile branch in the heat-shocked #3-1 plant (Fig. 2a; insert 3), a cauline leaf of the fertile branch was also assessed. As confirmed in the T2 generation, #3-1 plant siblings were homozygous for *gcs1* (Fig. 2b). Although a pre-recombined sequence containing the *GCS1* gene on the introduced T-DNA was detected from the non-heat-shocked fertile plant (Fig. 1a, amplified with primer set 1′), no amplification was detected from the heat-shocked, fully infertile branch (Fig. 2b). Interestingly, a partially fertile branch in the heat-shocked plant had the pre-recombined sequence (Fig. 2b), suggesting that heat-inducible Cre-*loxP* recombination was partially non-functional in the stem cell forming this branch. Such partially fertile branches were frequently observed in transgenic plants with heat-inducible fully infertile branches (Table 1). These results showed that the heat-shock-induced fully infertile phenotype was associated with complete loss of the *GCS1* gene in the transgenic plants. We also performed RT-PCR analysis of the infertile *gcs1/*− plant and confirmed that transcripts of *GCS1* were barely expressed in the *gcs1/*− plant, although slight expression of the region upstream of the T-DNA insert was detected (Fig. 2c).

We then checked the infertile phenotype by hand pollination experiments. The proportion of developed seeds was nearly 100 % when pollen from non-heat-treated #3-1 plants as well as heat-shocked or untreated wild-type plants was crossed to wild-type pistils (Fig. 2d, e, f). The proportion of developed seeds decreased significantly, to nearly 0 %, when crossed with pollen from the heat-shocked #3-1 plant (Fig. 2d, e, f). The same results were obtained using the untreated or heat-shocked plants of additional lines #16-10 and #19-12 (Fig. 2f). Normal female fertility of the heat-shocked #16-10 plant was confirmed by reciprocal crossing (Fig. 2g). Consequently, we achieved generation of male sterile plants without the *GCS1* gene.

### All pollen tubes from infertile plants contained *gcs1* sperm cells and showed the lowest fertilization rate as male mutant

**Fig. 3:**
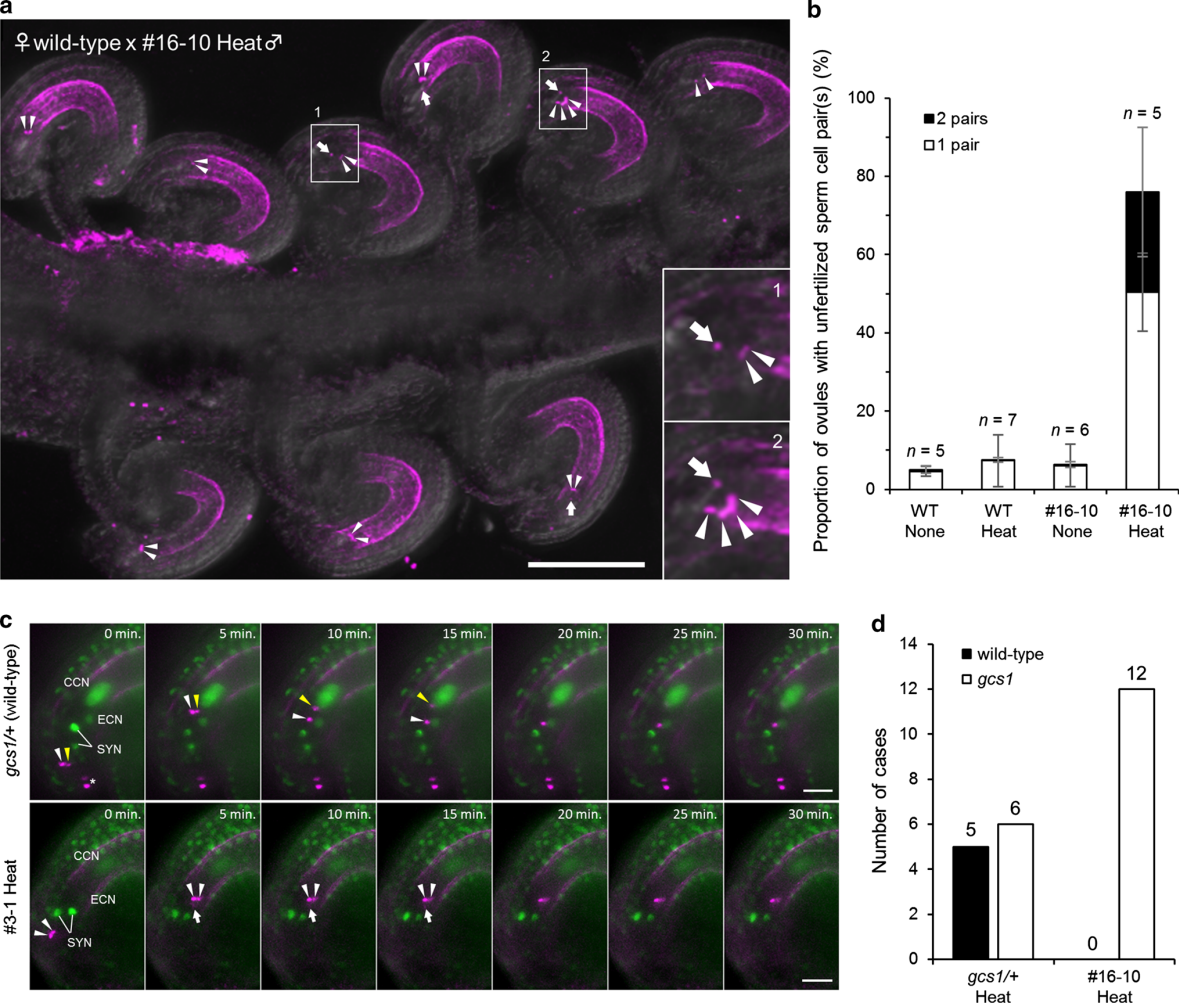
Observation of fertilization-defective sperm cells of the heat-shocked infertile plants. **a** Observation of the phenotype of *gcs1* sperm cells in vivo. The wild-type ovules crossed with the heat-shocked #16-10 plant were observed at 12 HAP by confocal laser microscopy. The maximum projections of optical sections are stacked. The *arrowheads* and *arrows* indicate the signals of the unfertilized sperm cell nuclei (HTR10-mRFP) and vegetative nuclei (H2B-tdTomato), respectively. *Insets* show magnifications of the ovules with one pair (1) or two pairs (2) of unfertilized sperm cells in areas marked by *squares* with *numbers*. *Scale bar*, 100 µm. **b** Quantitative analysis of the proportion of ovules with unfertilized sperm cell pairs in wild-type pistils at 24 HAP. The number of red fluorescent signals as shown in Fig. 3a was counted. For ovules crossed with wild-type pollen possessing *HTR10p::HTR10:mRFP* gene, zero, one to two, and three or more signals were considered as zero, one, and two pairs of unfertilized sperm cells, respectively. For ovules crossed with #16-10 pollen possessing both *HTR10p::HTR10:mRFP* gene and *RPS5Ap::H2B:tdTomato* gene with leaky expression, zero to one, two to three, and four to six signals were considered as zero, one, and two pairs of unfertilized sperm cells, respectively. The *white and black bars* indicate the proportion of one pair and two pairs of sperm cells, respectively. *Error bars* indicate standard deviation. *n*, Number of siliques examined. **c** Time series images (maximum projections of optical sections) of semi-in vivo fertilization. Sperm nuclei and nuclei of female gametophytic cells were labeled by mRFP and GFP, respectively. The upper image is a montage of double fertilization process of wild-type sperm cells observed with *gcs1/*+ plants. The *yellow and white arrowheads* indicate the sperm cell nuclei moving toward the central cell nucleus and the egg cell nucleus, respectively. The *asterisk* indicates sperm cells in the other pollen tube. The lower image is a montage of images in which released sperm cell nuclei (*arrowheads*) of a heat-shocked #3-1 plant did not move toward the egg cell and central cell nuclei. The *arrow* indicates the vegetative nucleus labeled by H2B-tdTomato, which was expressed after recombination induced by heat treatment. *Time* indicates elapsed time; 0 min indicates the last frame just before pollen tube discharge. *CCN* central cell nucleus, *ECN* egg cell nucleus, *SYN* synergid cell nuclei. *Scale bars*, 20 µm. **d** Histogram of the number of cases observed by semi-in vivo assay with heat-shocked *gcs1/*+ and T3 #16-10 plants. The *black and white bars* indicate the number of ovules in which sperm cells showed wild-type and *gcs1*-like phenotypes, respectively. The *numbers *over *each bar* indicate the number of ovules observed

We examined whether the heat-shocked infertile plants showed the defective *gcs1* sperm cell phenotype. The *gcs1* sperm cells were reported to have a defect in membrane fusion with the egg cell and central cell during double fertilization, resulting in sperm cells remaining within the ovule receiving a pollen tube (Mori et al. 2006). We observed signals of discharged sperm cells and a vegetative nucleus in ovules crossed with the heat-shocked infertile plants (Fig. 3a). Most of the ovules contained at least one pair of unfertilized sperm cells (Fig. 3a). We then counted the number of red fluorescent signals remaining in the ovules using non-heat-treated or heat-shocked wild-type and #16-10 plants as pollen donors. In the case of the heat-shocked #16-10 plant, two to three signals and four to six signals were considered to be one and two pairs of sperm cells, respectively. Heat-induced H2B-tdTomato expression was sometimes too weak to detect the vegetative nucleus. The proportion of ovules with red fluorescent signals was significantly increased in crosses using pollen from the heat-shocked #16-10, with one pair of sperm cells in 50.3 % and two pairs in 25.6 % of ovules, whereas the signals of unfertilized sperm cells were rarely observed in the ovules crossed with the wild-type and untreated #16-10 plants (Fig. 3b). Although about half of the remaining 24 % of ovules also had one signal, we were unable to conclude whether it was from unfertilized sperm cells or a vegetative nucleus due to the weakness of the signal. For the same reason, the estimated proportion of ovules with two sperm cell pairs may have been lower than the actual level.

To examine the phenotype of the *gcs1* sperm cells directly from the start of sperm cell release, we performed live-cell imaging of double fertilization by semi-in vivo fertilization assay (Hamamura et al. 2011). When crosses were performed with the pollen from a *gcs1/*+ plant, the behavior of wild-type sperm cells was observed as reported previously (Hamamura et al. 2011). The sperm cells labeled with HTR10-mRFP moved rapidly to the region between the egg cell and the central cell. Then, sperm nuclei moved toward the nuclei of their own fertilization targets (Fig. 3c, upper; Movie S1). In contrast, sperm cells of the heat-shocked #3-1 plant were discharged normally, but always remained motionless between the egg cell and the central cell (Fig. 3c, lower; Movie S2). In the case of *gcs1/*+, successful double fertilization occurred in about half of the ovules (5/11; Fig. 3d), and the *gcs1* sperm cell phenotype was observed in the other ovules (6/11; Fig. 3d). On the other hand, when the heat-shocked #16-10 plant was used as a pollen donor, the *gcs1* sperm cell phenotype was observed in all ovules (12/12; Fig. 3d). Finally, we concluded that the infertile plants induced by heat treatment were *gcs1/*− plants.

### Homozygous *gcs1* mutants can be obtained by infrequent fertilization of *gcs1* sperm cells

**Fig. 4:**
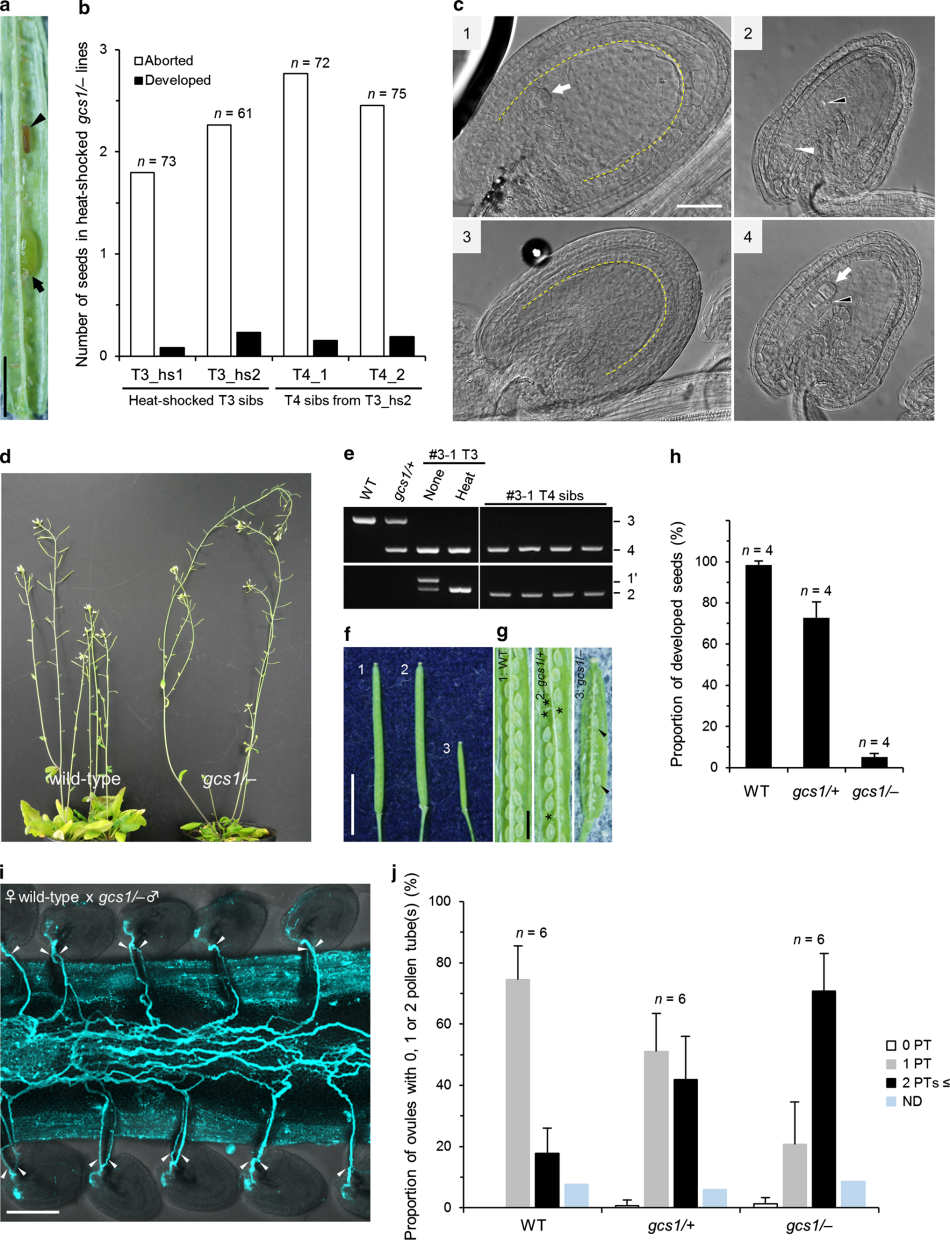
Analyses of seed development in heat-shocked infertile siliques and *gcs1* homozygous mutant plants obtained from the developed seeds. **a** Aborted (*arrowhead*) and developed (*arrow*) seeds generated in the heat-shocked infertile silique. *Scale bar*, 1 mm. **b** Average numbers of aborted and developed seeds per silique of heat-shocked infertile plants (T3_hs1, 2) and T4 plants. T4 siblings were obtained from the T3_hs2 line. *n*, Number of siliques examined. **c** Cleared wild-type ovules at 3 DAP crossed with wild-type (1) or *gcs1/*− (2–4) pollen. The *arrow* and *yellow dashed line* indicate the developed embryo and the outline of the developed endosperm, respectively. The *black and white arrowheads* indicate the unfertilized central cell nucleus and the egg cell, respectively. In the ovule accepting wild-type pollen shown in panel 1, normal embryo and endosperm development resulting from successful double fertilization were observed. In the ovules accepting *gcs1* pollen shown in panels 2, 3, and 4, unfertilized egg and central cells, the developed endosperm without embryo, and the developed embryo without endosperm were observed, respectively. *Scale bar*, 50 µm. **d** Comparison of the wild-type plant (*left*) with the infertile T4 plants grown from the developed seed in the heat-shocked infertile T3 *gcs1/*− plant (*right*). **e** Genomic PCR of the T4 siblings obtained from the heat-shocked T3 infertile plant. The *upper and lower images* represent the results of PCR using primer sets 3 and 4 (Fig. 1d) for endogenous *GCS1* and primer sets 1′ and 2 (Fig. 1a) for exogenous *GCS1*, respectively. The *left images* are positive controls of PCR using the genomic DNA used in Fig. 2b (for Heat, Heat_I was used). **f** Seven DAP wild-type siliques crossed with wild-type (1), *gcs1/*+ (2), or *gcs1/*− (3) plants. *Scale bar*, 5 mm. **g** Seed development in each silique shown in **f**. The *asterisks* indicate the undeveloped seeds in the silique pollinated by the *gcs1/*+ plant. The *arrowheads* indicate the developed seeds in the silique pollinated by the *gcs1/*− plant. *Scale bar*, 1 mm. **h** Quantitative analysis of the proportion of developed seeds in the seven DAP siliques crossed with the wild-type, *gcs1/*+, and *gcs1/*− plants. *Error bars* indicate standard deviation. *n*, Number of siliques examined. **i** Observation of pollen tube attraction of the wild-type ovules crossed with *gcs1/*− pollen by aniline blue staining. The pistil at 24 HAP was examined by confocal laser microscopy. The maximum projections of optical sections are stacked. The arrowheads indicate pollen tubes entering into the ovules. *Scale bar*, 100 µm. **j** Quantitative analysis of the proportion of ovules attracting zero, one, and two or more pollen tubes at 24 HAP. The numbers of pollen tubes entering into the wild-type ovules crossed with wild-type, *gcs1/*+, or *gcs1/*− pollen were counted by aniline blue staining. The *white*, *gray*, *and* *black bars* indicate the proportions of ovules with zero, one, and two or more pollen tubes, respectively. Note that while a few ovules attracting more than two pollen tubes were observed, it was difficult to provide an accurate count. *ND* not determined (*blue bar*). *Error bars* indicate standard deviation. *n*, Number of siliques examined

About 80 % (59/73, *n*  =  73 for T3_hs1; 48/61, *n*  =  61 for T3_hs2) of the short siliques of auto-pollinated infertile plants contained a few aborted and/or developed seeds (Fig. 4a, b). As other mutants defective in sperm cell function such as *gamete expressed 2* (*gex2*) and *kokopelli* were shown to produce aborted seeds resulting from single fertilization (Mori et al. 2014; Ron et al. 2010), we hypothesized that single fertilization of the egg cell or the central cell may be responsible for the aborted seeds in heat-inducible *gcs1* homozygous plants. By clearing ovules accepting the *gcs1* pollen tube, we observed ovules with only embryo or endosperm development, indicating single fertilization events (Fig. 4c), as observed in ovules fertilized by sperm cells complemented with partially functional GCS1 variants (Wong et al. 2010). Additionally, we found a few ovules with both embryo and endosperm in a silique, consistent with the presence of developed seeds (Fig. 4a). To determine whether the developed seeds were produced by successful double fertilization, we sowed the seeds on MS medium and analyzed the phenotype and genotype. The seeds germinated normally and grew into mature plants (Fig. 4d). Genomic PCR of the endogenous *GCS1* allele and the transgene indicated that most of the growing plants had no functional *GCS1* sequence similar to the heat-shocked T3 infertile parent (Fig. 4e), although some plants had either of the functional *GCS1* genes, probably due to unintended cross-pollination of fertile pollen grains (data not shown). In agreement with these results, the plants without the *GCS1* gene exhibited an infertile phenotype when crossed to wild-type pistils (Fig. 4f, g, h). The aborted and developed seeds were also observed in this T4 plant derived from the developed seeds in the heat-shocked T3 plant (Fig. 4b), suggesting that seed development in the *gcs1/*− plants was not due to the effect of heat treatment. Briefly, the *gcs1/*− plants could be maintained without heat treatment. Based on these results, we expected that *gcs1* sperm cells may occasionally fertilize at a low frequency.

It was reported that failure of fertilization by *gcs1* sperm cells in a *gcs1/*+ mutant caused attraction of a second pollen tube to an ovule accepting a *gcs1* pollen tube (Kasahara et al. 2012; Beale et al. 2012). In the case of *gcs1/*+ plants, 50 % of the ovules first accept a pollen tube containing *gcs1* sperm cells, and then ~80 % attract a second pollen tube for fertilization recovery (Kasahara et al. 2012; Beale et al. 2012). We postulated that the rate of ovules attracting a second pollen tube would increase by the use of *gcs1/*− plants because all of the ovules first receive a *gcs1*-pollen tube and most fail in double fertilization (Fig. 3). We counted the number of pollen tubes reaching the micropyle of wild-type ovules at 24 HAP by aniline blue staining using wild-type, *gcs1/*+, or *gcs1/*− plants as a pollen donor (Fig. 4i). The results indicated that only 17.9 % of the ovules attracted two or more pollen tubes when wild-type was used in the cross (Fig. 4j). In the case of *gcs1/*+, the proportion of ovules with two or more pollen tubes increased to 41.9 %, whereas 51.0 % of the ovules attracted one pollen tube. As expected, when *gcs1/*− was used as the pollen donor, 70.9 % of the ovules attracted two or more pollen tubes (Fig. 4j). These observations indicated that *gcs1/*− plants could facilitate the analysis of pollen tube attraction during fertilization recovery as a male mutant maximizing the number of ovules attracting a second pollen tube.

## Discussion

Studying reproductive process is of fundamental importance for understanding the underlying molecular mechanisms and increasing crop production by manipulation of reproductive genes in angiosperms. One of the major problems limiting the functional analysis of reproductive genes is the inaccessibility of fully defective homozygous mutants of genes involved in the reproductive process, which cannot be obtained naturally due to gametophytic dysfunction. In this study, we succeeded in generating *gcs1* homozygous plants in which all male gametophytes contained fertilization-defective sperm cells, which cannot be obtained by self-crossing of *gcs1/*+ mutants (Mori et al. 2006). The homozygous mutant-inducible system developed here will be useful for investigating the functions of well-known and unknown factors in addition to GCS1 during the reproductive process. A sequence of interest can be introduced easily into the pMDC99/rescue vector by LR reaction, which is unrestricted by restriction enzyme sites for cloning into the vector, to rescue mutants (Fig. 1a). Our method is applicable to essential genes that function specifically in male and/or female gametophytes. As all gametophytes of the homozygous mutant plants carry mutant gametes, analyses of fertilization-defective mutants will become more efficient, as shown in this study (Figs. 3, 4). For example, the efficiency of observation of the *gcs1* phenotype was doubled using homozygous *gcs1* mutant for semi-in vivo fertilization assay (Fig. 3d).

Identification of novel fertilization-related factors and analyses of known factors are necessary to understand the molecular mechanisms underlying double fertilization in *Arabidopsis*. To explore novel genes involved in double fertilization, comparative transcriptomics or proteomics analyses using wild-type and purely male/female mutant gametes will be informative. Although mutants showing a strong phenotype may be required for the detection of important factors in such differential analyses, it is difficult to obtain homozygous mutants (Mori et al. 2006; von Besser et al. 2006; Durbarry et al. 2005). Therefore, an efficient method to artificially produce homozygous mutants would be important for such studies. Our heat-inducible system will be a useful means of addressing the difficulty of generating homozygous mutants for various genes. For example, application of the system to generate the *duo1* mutant, which shows defects in sperm cell specification and fertilization (Durbarry et al. 2005; Rotman et al. 2005), may accelerate identification of factors essential for sperm cell function, including cell–cell communication among male and female gametes during double fertilization. DUO1 is a transcription factor that regulates expression of many genes, including *GCS1* and downstream transcription factors specifically expressed in sperm cells (Brownfield et al. 2009; Borg et al. 2011). Comparative transcriptomics/proteomics analyses using isolated *duo1* sperm-like cells from homozygous mutants obtained after application of our system would be a good strategy to identify the molecular mechanism of double fertilization, which still remains largely unknown. As several methods for mass isolation of male gametes of *A. thaliana* (Borges et al. 2008, 2012) and transcriptomics/proteomics analyses using sperm cells in various plants have been reported (Borges et al. 2008; Abiko et al. 2013; Zhao et al. 2013), it would be feasible to conduct large-scale analyses in the future.

We observed a very few developed seeds that grew into *gcs1/*− plants (Fig. 4d), which might result from the fertilization of the *gcs1* sperm cells and the egg and central cell (Fig. 4c). This occasional fertilization was not detected previously using heterozygous mutants of *gcs1* (Mori et al. 2006) or *hap2* (von Besser et al. 2006) alleles, although the male transmission of *hap2* allele was suggested at a very low frequency (0.7 %; Johnson et al. 2004). GCS1/HAP2 is also an indispensable factor for fertilization in *Chlamydomonas* and *Plasmodium* (Liu et al. 2008; Hirai et al. 2008). It is possible that the truncated GCS1 protein with extracellular and transmembrane domains is responsible for this occasional fertilization, which is suggested by the expression of *gcs1* transcript upstream of the T-DNA (Fig. 2c). This is supported by the observation that modified GCS1 variants rescued the *gcs1/hap2* phenotype at different levels (Mori et al. 2010; Wong et al. 2010). To examine the possibility that the partial function of the truncated GCS1 protein is responsible for the low-frequency fertilization, analysis using other alleles, such as *hap2* allele (von Besser et al. 2006), would provide insights into this possibility. The T-DNA insertion site of the *hap2* allele is earlier in the *GCS1* gene than *gcs1* allele we used in this study. Generation of homozygous mutants by heat-inducible recombination is useful for examining whether a mutant allele is fully infertile. In this study, using this strategy, rate of successful fertilization and seed development by the *gcs1* sperm cells was estimated to be 0.16–0.46 % (0.08–0.23 seeds per silique with 50 ovules; Fig. 4b). As the *gcs1* homozygous mutant could be maintained by self-pollination, seeds of this mutant will be useful for plant fertilization research.

We also showed that the fertilization recovery system worked strictly in over 70 % of the ovules first accepting *gcs1* sperm cells (Fig. 4h). Remarkably, not all of the ovules accepting a *gcs1* pollen tube attracted a second pollen tube, consistent with a previous report using *gcs1/*+ (Kasahara et al. 2012). These results suggest that polytubey block, including the inactivation of the remaining synergid cell mediated by ethylene signaling and unidentified dual control from the egg cell and central cell (Völz et al. 2013; Maruyama et al. 2013), may be induced partially just by the reception of a pollen tube. On the other hand, we observed ovules attracting more than two pollen tubes corresponding to a previous report (Beale et al. 2012). However, we could not show whether reception of more than two pollen tubes was completed and how attraction of multiple pollen tubes occurred by the ovule with two synergid cells. The *gcs1/*− plants generated here may facilitate detailed analyses of the regulation of pollen tube attraction and polytubey block during fertilization recovery, including the secretory pattern of the pollen tube attractant LURE from the remaining synergid cell (Takeuchi and Higashiyama 2012). The *gcs1/*− plants are also expected to be useful for determining the changes in the ovule occurring after sperm cell release preceding double fertilization, which may include the secretion of EC1 peptides from the egg cell to activate the sperm cells (Sprunck et al. 2012). Analyses of the egg cell and central cell that have been exposed to the environment just before double fertilization are expected to be applied, in combination with methods for the isolation of the female gametes (for a review, see Wuest et al. 2013) from the ovules accepting the *gcs1/*− pollen. Comparative transcriptomics/proteomics between ovules receiving *gcs1* sperm cells and unfertilized ovules may uncover the alterations of gene expression and protein expression that could separate the reproductive process before from that after sperm cell release.

### **Author contribution statement**

SN and HT designed the experiments. SN carried out most of the experiments. HT carried out in vivo phenotype analysis with SN. TH supervised the project. SN wrote the manuscript, and HT and TH edited it.

## Electronic supplementary material

Below is the link to the electronic supplementary material.

Supplementary material 1 (PDF 118 kb)

Supplementary material 2 (MPG 110 kb)

Supplementary material 3 (MPG 298 kb)
