# Supplementary material for: Generation of a homozygous fertilization-defective gcs1 mutant by heat-inducible removal of a rescue gene
Source: Plant Reprod. 2015 Feb 12;28(1):33–46. doi: 10.1007/s00497-015-0256-4 (PMC4333230; doi:10.1007/s00497-015-0256-4)
Supplement: Supplementary file 1 — Supplementary material 1 (PDF 118 kb) [file 497_2015_256_MOESM1_ESM.pdf]

Supplementary material

Plant Reproduction

Title: Generation of a homozygous fertilization-defective *gcs1* mutant

Shiori Nagahara[1], Hidenori Takeuchi[1,2], Tetsuya Higashiyama[1,2,3,*]

[1]Division of Biological Science, Graduate School of Science, Nagoya University, Furo-cho, Chikusa-ku, Nagoya, Aichi 464-8602, Japan.

[2]JST ERATO Higashiyama Live-Holonics Project, Nagoya University, Furo-cho, Chikusa-ku, Nagoya, Aichi 464-8602, Japan.

[3]Institute of Transformative Bio-Molecules (WPI-ITbM), Nagoya University, Furo-cho, Chikusa-ku, Nagoya, Aichi 464-8602, Japan.

[*]Corresponding author: email, higashi@bio.nagoya-u.ac.jp

Movie S1 Live-cell imaging of double fertilization in the ovule accepting wild-type sperm cells of the *gcs1/+* plant. Sperm nuclei and nuclei of female gametophytic cells were labeled by mRFP and GFP, respectively. The arrowheads indicate the sperm cell nuclei moving toward the central cell nucleus and the egg cell nucleus. Time indicates elapsed time; 0 min indicates the last frame just before pollen tube discharge. See also Fig. 3c (upper)

Movie S2 Live-cell imaging of fertilization defect in the ovule accepting *gcs1* sperm cells of the heat-shocked #3-1 plant. Sperm nuclei and nuclei of female gametophytic cells were labeled by mRFP and GFP, respectively. The arrowheads indicate the sperm cell nuclei moving toward the central cell nucleus and the egg cell nucleus. Time indicates elapsed time; 0 min indicates the last frame just before pollen tube discharge. See also Fig. 3c (lower)

Table S1 Primers used for construction, genomic PCR and RT-PCR

Name	Primer sequence (5' to 3')	Used for
*Asc*I_*Hsp*_F	ggcgcgccTCTAGATAGTCAGCC	amplifing *Hsp-Cre-NosT*
*Kpn*I_*NosT*_R	ggtaccCCCGATCTAGTAACATAG	
*Sbf*I_*loxP*_F1	cctgcaggATAACTTCGTATAGCATACATTATACG	amplifing *loxP-H2B-tdTomate-NosT*
*Pme*I_*NosT*_R	gtttaaacGATCTAGTAACATAGATG	
*RPS5Ap*_F+*Spe*I	tggactagtGATCCCTCAACTTTTGATTCG	amplifing *RPS5Ap-loxP*
*Spe*I_*loxP*_R1	actagtATAACTTCGTATAATGTATGCTATACGAAG	
*GCS1*_-1002F+*Xba*I	ctagtctagaAGCAGAGCACATCTTATC	amplifing *GCS1p-GCS1-NosT*
*GCS1*_R+*Sac*I+*Nco*I+	caccccatgggagctcTTAACTCTCACGTAGTC	
*Nco*I_*NosT*_F	ccatggCGTTCAAACATTTGGC	
*Not*I_*NosT*_R	gcggccgcGATCTAGTAACATAG	
*RPS5A*_-1572F	AACGATCTTCAGGTGATCTTC	genotyping of heat-shocked T1 plants (primer sets 1 and 2 in Fig. 1a)
*H2B*_+133R	CCTTTGGTGGCAATTTCTTC	
*GCS1*_-1R+*Xba*I	tgctctagaTTTCTCTCTCACGGAGACG	
*GCS1*_*gcs1* resc._genotyping_R	CTTATGCTGGAAGATTGAAC	genotyping of heat-shocked T3 and T4 plants (primer 1' in Fig. 1a)
*GCS1*_*intron16*_F	CTCGAACTAACGGTTTTGTTTTCAG	*gcs1* genotyping (primer sets 3 and 4 in Fig. 1c)
*GCS1*_*3'UTR*_R	GCTTCACAATCCAGTTGGGATTC	
LBb1.3	ATTTTGCCGATTTCGGAAC	
*GCS1*_RT-PCR_2154F	GCACTTACACAATTCGGAGTTG	RT-PCR (*GCS1*_up in Fig. 2c)
*GCS1*_RT-PCR_2786R	CTGGAATTTGAAATGGATTAGTAAC	
*GCS1*_RT-PCR_2996F	TCTTCTCCTCGCTCTCTTCC	RT-PCR (*GCS1*_int in Fig. 2c)
*GCS1*_RT-PCR_3358R	GAGTCGCTGTGGTCACGTTC	
*TUB4*_690F	TCTCTGTGCATCAGCTTGTC	RT-PCR (*TUB4* in Fig. 2c)
*TUB4*_1056R	GCTGTGATCCTCTCGATGTC	

The additional sequences for restriction enzyme sites are represented in lower-case letters
